# Protein Detection with Aptamer Biosensors

**DOI:** 10.3390/s8074296

**Published:** 2008-07-23

**Authors:** Beate Strehlitz, Nadia Nikolaus, Regina Stoltenburg

**Affiliations:** UFZ - Helmholtz Centre for Environmental Research, UBZ, Permoserstr. 15, 04318 Leipzig, Germany; E-Mails: nadia.nikolaus@ufz.de; regina.stoltenburg@ufz.de

**Keywords:** aptamer, protein, biosensor, SELEX

## Abstract

Aptamers have been developed for different applications. Their use as new biological recognition elements in biosensors promises progress for fast and easy detection of proteins. This new generation of biosensor (aptasensors) will be more stable and well adapted to the conditions of real samples because of the specific properties of aptamers.

## 1. Introduction

There is a high demand for convenient methodologies for detecting and measuring the levels of specific proteins in biological and environmental samples because their detection, identification and quantification can be very complex, expensive and time consuming. Biosensors are interesting tools offering certain operational advantages over standard photometric methods, notably with respect to rapidity, ease-of-use, cost, simplicity, portability, and ease of mass manufacture. Biosensors have been developed for more than 25 years now, and have been commercialized for some special applications like blood glucose and lactate measurement or bioprocess control, amongst others. However, they have not entered the market as much as expected, which is caused by several reasons. One reason is the instability of the biological recognition element of the biosensor (e.g. enzymes, cells or antibodies). Aptamers, which are ssDNA or RNA oligonucleotides, can bind to their targets due to their specific three dimensional structures; they offer specific properties which favor them as new biorecognition elements for biosensors. In particular their outstanding and modifiable stability and their regenerative target binding promise the development of a new biosensor generation. Aptasensors [1] open up new vistas for the detection of analytes which are not accessible to easy and fast detection methods until now.

Until now, proteins are detected mostly by antibodies in analytical formats like ELISA, immunobead assay, western blotting, microarrays and also biosensors. Aptamers are equal to monoclonal antibodies concerning their binding affinities, but furthermore, they provide decisive advantages. They are more resistant to denaturation and degradation, their binding affinities and specificities can easily be manipulated and improved by rational design or by techniques of molecular evolution, and they can be modified with functional groups or tags that allow covalent, directed immobilization on biochips, resulting in highly ordered receptor layers [2]. Aptamers can distinguish between chiral molecules and are able to recognize a distinct epitope of a target molecule [3, 4]. In principle, aptamers can be selected for virtually any desired target, even non-immunogenic or toxic proteins, because they are produced in vitro by an evolutionary method called SELEX (systematic evolution of ligands by exponential enrichment) [5, 6], without the constraints imposed by having to be selected or produced in a living organism. The selection of ligands beyond natural systems emanates from a chemically produced oligonucleotide library with the big variety of, e.g., 10^15^ different oligonucleotides. The number of variation depends on the length of the variable region. With a variable region of 25 oligonucleotides, there are, theoretically, 4^25^ (≅ 10^15^) different oligonucleotide sequences possible. The big variety of the oligonucleotide library and the amplification steps of target-binding oligonucleotides during the selection process considerably facilitates the selection of ligands with highest affinity compared to natural selection [7]. Moreover, the SELEX process can be carried out under conditions akin to those used in the assay for which the aptamer is being developed. As a consequence, the aptamer will maintain its structure and will function in the final assay. Especially, the aptamer will not dissociate or otherwise change its characteristics, which can be a problem with antibodies [8]. The SELEX conditions can be further modified to direct the selection to aptamers with desired features. This is in contrast to the classical production of antibodies, where it is not possible to influence such parameters which therefore leaves the resulting bioreceptors (antibodies) limited to physiological conditions [9]. Another advantage of using aptamers instead of antibodies for biosensing applications is the fact that non-specific adsorption phenomena are usually less pronounced on nucleic acid derivated surfaces as compared to protein derivated ones [10].

Although aptamers have been developed for all classes of targets ranging from small molecules to large proteins and even cells, proteins seem to be the biggest group of target molecules. In principle, it should be possible to generate aptamers for virtually every protein target. However, it is striking that there is only a small range of proteins that are detected using aptasensors (cf. Table 1). This review gives an overview of recent developments and applications of aptamer biosensors for protein detection.

## 2. Biosensor

**Figure 1.**
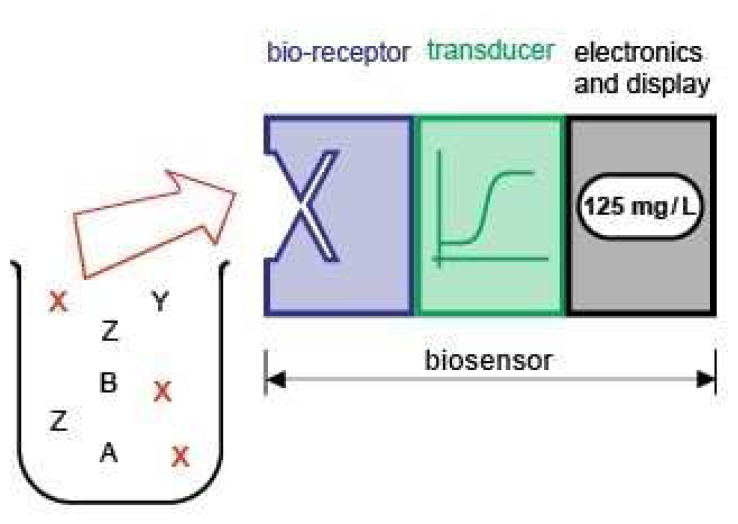
Biosensor principle. A biosensor consists of a bioreceptor for the specific detection of the respective analyte in spatial contact to a transducer for converting the signal into an electrically manageable format and a signal processing unit.

As per definition of IUPAC, a biosensor is an integrated receptor-transducer device, which is capable of providing selective quantitative or semi-quantitative analytical information. The biosensor consists, on the one hand, of a biological recognition element, which acts upon a biochemical mechanism, and, on the other hand, of a transducer relying on electrochemical, mass, optical or thermal principles (Figure (1)). The characteristic trait of a biosensor is the direct spatial contact between the biological recognition element (or bioreceptor) and the transducing element [11]. Typical bioreceptors in biosensors are enzymes, antibodies, microorganisms, and nucleic acids. Aptamers are a new promising group of bioreceptors, because of their outstanding selectivity, sensitivity and stability, the reproducibility of the target binding reaction, their production by chemical synthesis ensuring a constant lot-to-lot quality, and the ease of regeneration of aptamer derivated surfaces.

## 3. Protein biosensor detection principles based on aptamers

Biosensors for protein detection mainly involve antibodies, but lately, also aptamers as biological recognition elements in the case of specific detection and enzymes in the case of total protein detection.

Aptamers can rival antibodies in a number of applications. Aptamers are very small in size (ca. 30 to 100 nucleotides) in comparison to other biorecognition molecules like antibodies or enzymes. This allows efficient immobilization at high density. Therefore, production, miniaturization, integration, and automation of biosensors can be accomplished more easily with aptamers than with antibodies. Once selected, aptamers can be synthesized with high reproducibility and purity. DNA aptamers are usually highly chemically stable enabling reusability of the biosensors. In contrast, RNA aptamers are susceptible to degradation by the endogenous ribonucleases typically found in cell lysates and serum. Therefore, biosensors using RNA aptamers as bio-recognition elements can be used only for single shot measurements in biological surroundings [12]. In order to circumvent this problem, modifications of the 2′ positions of pyrimidine nucleotides with amino/fluoro groups have been introduced [13, 14]. Another possibility is the use of RNase inhibitors [15].

The significant conformational change of most aptamers upon target binding offers great flexibility in the design of biosensors with high detection sensitivity and selectivity. Protein targets with their high structural complexity allow aptamer binding by stacking interactions, shape complementary, electrostatic interactions, and hydrogen bonding. Moreover, in principle, proteins can present more than one binding site for aptamers, allowing the selection of a pair of aptamers binding to different regions of the target and enabling sandwich-assay based biosensors.

### 3.1. Electrochemical aptasensors

Electrochemical transduction of biosensors using aptamers as bioreceptors include methods like Faradaic Impedance Spectroscopy (FIS), differential pulse voltammetry, alternating current voltammetry, square wave voltammetry, potentiometry or amperometry.

In principle, it can be differentiated between either a positive or negative readout signal, i.e. an increase or a decrease of response following upon receptor-target interaction, cf. [10].

Xu et al. demonstrated an electrochemical impedance spectroscopy detection method for aptamer-modified array electrodes as a promising label-free detection method for IgE [16]. They compared DNA aptamer based electrodes with anti-human IgE antibody based electrodes and found lower background noise, decreased nonspecific adsorption, and larger differences in the impedance signals due to the small size and simple structure of the aptamers in comparison to the antibody [16].

Impedance sensors allow the real-time monitoring of the sensor signal and can give rise to kinetic aspects of the ligand-analyte interaction [17]. Schlecht et al. have compared an RNA aptamer and an antibody for thrombin detection by use of a nanometer gap-sized impedance biosensor. They found that both ligands showed equal suitability for the highly specific detection of their analyte. Their device has a multiplexer-approach enabling the parallel readout of five sensor elements. This opens up the possibility to use reference sensors for the elimination of background signals and simultaneous detection of different analytes by immobilizing their respective ligands on separate electrodes [17].

For impedance methods, usually a negative readout signal can be found in consequence of an increase in electron transfer resistance. However, Rodriguez et al., 2005 described the set-up of an impedance-based method exhibiting a positive readout signal [18] making use of the change of surface charge from negative to positive upon target protein binding (at proper pH).

A very similar approach, also depending on electrostatic interactions, was made by Cheng et al., 2007. A DNA aptamer for lysozyme was immobilized on gold surfaces by means of self-assembly and [Ru(NH_3_)_6_]^3+^ bound to the DNA phosphate backbone via electrostatic interaction. The surface density of aptamers can be determined by measuring the [Ru(NH_3_)_6_]^3+^ reduction peak height in the cyclic voltammogram. Upon target binding of lysozyme to the aptamers, the surface bound [Ru(NH_3_)_6_]^3+^ cations are released. This can be detected as a decrease in the integrated charge of the reduction peak [19].

The hindrance of the redox reaction of K_3_Fe(CN)_6_ on a gold surface due to an increased density of the covering layer by binding of the immobilized DNA aptamer with its target thrombin [20] was used as signal for the binding reaction. The signal was measured by cyclic voltammetry. The aptasensor for thrombin is reusable and allows measurements in the relevant analytical range for clinical applications (cf. Table 1) [20].

Another label free method is to use intercalators that bind to double stranded regions of the aptamer. If these regions are close enough to the electrode, the intercalators can serve as reporters. Upon binding and the consecutive conformational changes, the intercalator can be released producing a negative response. An example is described in [21] where an aptamer for thrombin was immobilized on a gold electrode. Methylene blue (MB) intercalates into a double strand region and will be released upon target binding because of the conformational change of the aptamer. The MB cathodic peak current in the differential pulse voltammogram decreases with increasing thrombin concentration.

These techniques described above are label-free, that is, neither the bioreceptor nor the target has to be covalently labeled with indicator molecules and this therefore omits a further step in the production process of the sensor. In contrast, many electrochemical aptasensors rely on the labeling of the bioreceptor with a reporter unit.

For example, aptamers can be labeled at both ends. At one end, a moiety for immobilization at the surface is tethered to the aptamer and at the other end, the reporter. The electrode surface is then covered with a layer of those aptamers. Upon target binding, the mobility of the aptamer and/or the density of the layer are altered due to beacon-like conformational changes. This results in a smaller or greater distance of the reporter unit from the electrode leading to an increased or decreased electron transfer, respectively [22, 23].

Sandwich assays rely on the possibility that more than one aptamer can be generated for one protein target. One aptamer, attached to the sensor surface, binds the target at one epitope. The second aptamer, directed to a different epitope is labeled with the reporter, e.g., (PQQ) glucose dehydrogenase. Binding of the second aptamers to the target brings the reporter in proximity to the sensor surface. After a washing step, the binding is detected (in this case by amperometry after addition of glucose as a substrate for (PQQ) glucose dehydrogenase) leading to a positive readout signal via the redox mediator 1-methoxyphenazine methosulfate [24].

### 3.2. Optical aptasensors

Optical transduction methods in aptasensors comprise, for example, the utilization of surface plasmon resonance, evanescent wave spectroscopy, as well as fluorescence anisotropy and luminescence detection.

Surface plasmon resonance (SPR) and evanescent wave based biosensors rely on the change of optical parameters upon changes in the layer closest to the sensitive surface. Since the binding of, for example, proteins to a receptor layer of those biosensors changes the refractive index of the layer, the event of binding can be detected and quantified in a label free way.

Examples for the use of surface plasmon resonance biosensor detection of the respective target binding to the bioreceptor – the aptamer (in most cases thiolated for the immobilization at gold surfaces by self-assembly) – can be found in [25], [26] and [27]. Thrombin was captured by a DNA aptamer immobilized at Biacore™ chips. Several parameters like incubation time, incubation temperature effect of immobilization orientation etc. were extensively studied and optimized [25]. IgE was captured by a DNA aptamer with a detection limit of 2 nM and a linear range of detection from 8.4 to 84 nM using a combination of the methods of SPR and fixed-angle imaging [26]. HIV-1 Tat protein was captured by an RNA aptamer with a linear detection range from 0 to 2.5 ppm using a Biacore X™ instrument. Due to the inherent sensitivity of RNA to nucleases, all instrumentation was freed from RNases prior to preparation of the sensor chips and measurements [27].

**Figure 2.**
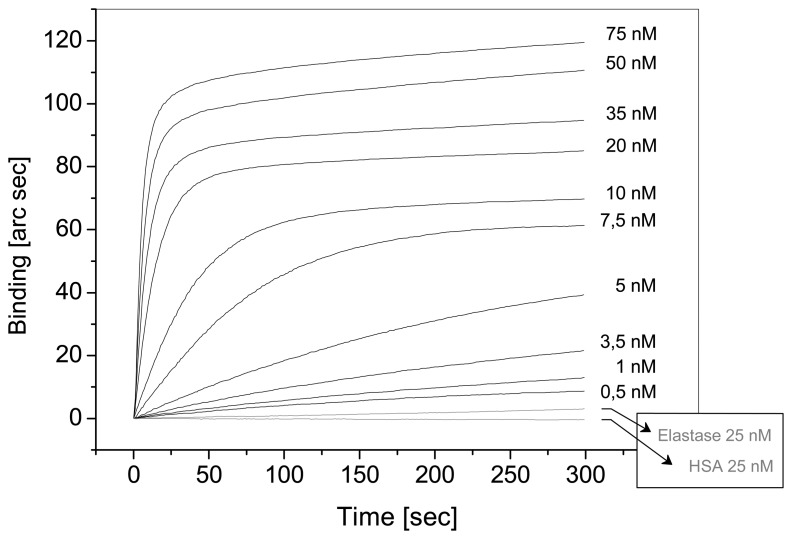
Binding of increasing amounts of human thrombin (0.5 … 75 nM) to the immobilized 3′ biotinylated anti-thrombin aptamer (15 nt, G-quartet), measured by use of the IAsys-system. Conditions: measurement in TA-buffer (20 mM TRIS-HCl, pH 7.4, 140 mM NaCl, 5 mM KCl, 1 mM CaCl_2_, 1 mM MgCl_2_), time 5 min, Negative controls: Elastase and HSA (25 nM each).

**Figure 3.**
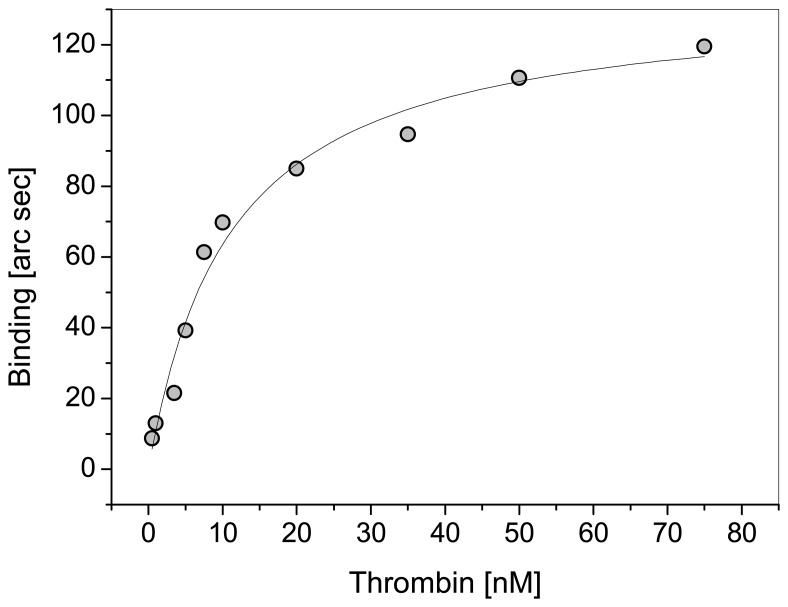
Saturation curves generated from results in Fig.2. Each point represents the measuring signal for one thrombin concentration after 5 min measuring time. The fitted curve was used for the determination of K_d_ by nonlinear regression analysis (K_d_ = 11.06 nM).

We have constructed a thrombin aptasensor (unpublished results) by immobilisation of the anti-thrombin aptamer, selected by Bock et al. 1992 [28], via biotin on a streptavidin modified surface of an IAsys cuvette. IAsys (Neosensors Ltd., UK) is a real time evanescent wave biosensor. The binding of different concentrations of thrombin (0,5 nM – 75 nM) in TA-buffer (20 mM TRIS-HCl, pH 7,4, 140 mM NaCl, 5 mM KCl, 1 mM CaCl2, 1 mM MgCl2) was assayed. Elastase and HSA (25 nM each) were used as negative controls. The results (binding curves) are shown in Figure (2). The saturation curve was constructed from the binding curves (measuring time 5 min, Figure (3)). The dissociation constant K_d_ was determined by nonlinear regression analysis (K_d_ = 11.06 nM) and is in good accordance with published results in the range of 5 to 300 nM detected with different methods [25, 28, 29].

Abrin toxin is highly toxic to eukaryotic cells with possible applications as an immunotoxin in cancer chemotherapy and as a potential biological warfare agent. A promising rapid and specific detection method is a DNA aptamer biosensor based on luminescence change detection caused by a molecular light switching intercalator [Ru(phen)_2_(dppz)]^2+^, which binds into duplex nucleic acid domains of the folded aptamer, emitting luminescence. Conformation changes of the aptamer upon target binding result in a significant target-dependent luminescence change [30].

An aptamer array sensor was developed for the parallel detection of four analytes (thrombin and the cancer associated targets inosine monophosphate dehydrogenase II – IMPDH, vascular endothelial growth factor – VEGF, basic fibroblast growth factor – bFGF). The transduction principle here is based on fluorescence polarization. All four immobilized aptamers (DNA for thrombin, RNA for the others) showed highly specific responses to their protein targets, even in a complex biological solution [12].

### 3.3. Mass sensitive aptasensors

Microgravimetric methods on piezoelectric quartz crystals base on the change of the oscillation frequency of the crystal upon mass change at its surface due to receptor-target binding (quartz crystal microbalance, QCM). This change of oscillation frequency is the signal that is detected. With this method, a label-free detection of the target is possible. However, the use of “weight labels” – e.g. aptamer functionalized Au nanoparticles – for the amplification of the binding reaction on the QCM surface seems useful [31].

Quartz crystals were coated with gold layers and streptavidin was subsequently immobilized. Biotinylated aptamers were then added and used as the receptor layer. DNA aptamers were used for the detection of IgE with a detection limit of 100 μg/L and a linear detection range from 0 to 10 mg/L. HIV-1 Tat protein was detected using RNA aptamers as receptors. Detection limits of 0.25 ppm and 0.65 ppm with linear detection ranges of 0 – 1.25 ppm and 0 – 2.5 ppm, respectively, were achieved [15, 27].

### 3.4. Potentiometric aptasensors

Potentiometric sensors are based on the measurement of a difference in potential between working and reference electrode caused by a difference in analyte concentration. Field effect transistors belong to the class of potentiometric sensors. Carbon nanotube field-effect transistors (CNT-FETs) are among the most promising candidates to possibly succeed to CMOS (complementary metal–oxide– semiconductor) technology by further miniaturization. The semiconducting behavior of CNTs is the main reason for the endeavor to build CNT-FETs.

Aptamer-modified CNT-FETs for the detection of IgE were constructed and compared to CNT-FET biosensors based on a monoclonal antibody (mAb) against IgE [32]. 5′-amino-modified 45-mer aptamers and IgE-mAb were immobilized on the CNT channels, respectively. The amount of the net source-drain current increased in dependence of the IgE concentration after IgE introduction on the aptamer-modified CNT-FETs. The detection limit of 250 pM and linear dynamic range of 250 pM to 20 nM was determined. The IgE-mAb sensor showed only a small change of the net source-drain current at 0.2 and 1.8 nM IgE. The aptamer-modified CNT-FETs displayed a better performance for IgE detection under similar conditions than the monoclonal antibody based CNT-FET [32].

## 4. Aptamer biosensors for protein detection

In the following table (Table 1), aptamer biosensors for different protein targets are presented and listed according to the kind of nucleic acid of the aptamer (DNA or RNA), the transduction mode and their reporter units (mediators, enzymes, dyes, etc.). Also, the achieved detection limits and linear detection ranges are listed.

## 5. Conclusions

The use of aptamers as new biological receptors can accelerate the development of biosensors of practical relevance. Because of their exceptionally high stability, selectivity and sensitivity, aptasensors have the potential to overcome the lacking functional and storage stability of most biosensors (besides some exceptions like glucose and lactate biosensors very well established on the market). This review shows that a big variety of biosensor principles (e.g. electrochemical, optical, mass sensitive) is available for the use of aptamers as biological receptors. However, only for a few proteins (thrombin, lysozyme, IgE and some others) aptasensors were described. The more aptamers for proteins will be developed and characterized, the more aptasensors will be developed in the future.

## Figures and Tables

**Table 1.** Aptamer biosensors for proteins.

Target Protein	Aptamer	Type of Sensor, Reporter Unit	Detect. Limit, Linear Range	Ref
Thrombin	DNA beacon	ec, differential pulsevoltammetry, methyleneblue intercalator	11 nM 0 … 50.8 nM	[21]
Thrombin	DNA	ec, impedancespectroscopy, [Fe(CN)6]^3-/4-^	2 nM 5 … 35 nM	[20]
Thrombin	DNA thiolated/biotinylated	ec, differential pulsepolarography, p-nitroaniline/peroxidase/HRP	80 nM/ 3.5 nM n.s.	[33]
Thrombin	DNA labeled with methylene blue	ec, alternating currentvoltammetry, methylene blue	n.s. n.s. (logarithmic dependence)	[23]
Thrombin	DNA labeled with pyrroquinoline quinone glucose dehydrogenase (PQQ)GDH, sandwich assay	ec, amperometry, glucose;single shot sensor	10 nM 40 … 100 nM	[24]
Thrombin	DNA ferrocene labeled	optical combined with ec(cyclic voltammetry), eSPR/ ec, amperometry with co-immobilized microperoxidase	n.s. n.s.	[22]
Thrombin	DNA thiolated/ biotinylated	optical, SPR (Biacore™)	n.s. n.s.	[25]
Thrombin/ Lysozyme	n.s., thiolated	ec, square wave stripping voltammetry	0.5 pM (20 … 500 ng/L)^1^	[34]
Lysozyme	DNA	ec impedance spectroscopy, [Fe(CN)_6_]^3-/4-^		[18]
Lysozyme	DNA	ec, [Ru(NH_3_)_6_]^3+^ cv peak decrease with target binding	0.5 μg/ml 0.5 … 50 μg/ml	[19]
IgE	DNA thiolated	optical, SPR	2 nM 8.4 … 84 nM	[26]
IgE	DNA biotinylated	mass sensitive, QCM	100 μg/L 0 … 10 mg/L	[35]
IgE	DNA	carbon nanotube FET	250 pM 250 pM … 20 nM	[32]
IgE	DNA	ec impedance spectroscopy, array	0.1 nM 2.5 … 100 nM	[16]
HIV-Tat protein	RNA biotinylated	optical, SPR/ mass sensitive, QCM	n.s./ 0.25 ppm 0… 2.5 ppm/ 0 … 1.25 ppm	[27]
HIV-Tat 1 protein	RNA biotinylated	mass sensitive, QCM	0.65 ppm 0… 2.5 ppm	[15]
Abrin toxin	DNA	optical, luminescence, molecular light switching intercalator	1 nM 1 … 400 nM	[30]
Thrombin, bFGF, IMPDH, VEGF	RNA, DNA, fluorescently labeled	optical array, fluorescence polarization anisotropy	n.s.	[12]

ec electrochemical

n.s. not specified

1 given as “analytically useful concentration dependence”

## Abbreviations

bFGF: Basic fibroblast growth factor
CNT-FET: Carbon nanotube field-effect transistor
CMOS: Complementary metal–oxide–semiconductor
DNA, ssDNA: Desoxyribonucleic acid, single stranded desoxyribonucleic acid
ELISA: Enzyme linked immunosorbent assay
FET: Field effect transistor
FIS: Faradaic Impedance Spectroscopy
HSA: Human serum albumin
IMPDH: Inosine monophosphate dehydrogenase
IUPAC: International Union of Pure and Applied Chemistry
K_d_: Dissociation constant
mAb: Monoclonal Antibody
MB: Methylene Blue
QCM: Quartz crystal microbalance
RNA: Ribonucleic acid
RNAse: Ribonuclease
SELEX: Systematic evolution of ligands by exponential enrichment
SPR: Surface plasmon resonance
VEGF: Vascular endothelial growth factor
